# iVision HHID: Handwritten hyperspectral images dataset for benchmarking hyperspectral imaging-based document forensic analysis

**DOI:** 10.1016/j.dib.2022.107964

**Published:** 2022-02-16

**Authors:** Ammad Ul Islam, Muhammad Jaleed Khan, Muhammad Asad, Haris Ahmad Khan, Khurram Khurshid

**Affiliations:** a Artificial Intelligence and Computer Vision lab (iVision), Institute of Space Technology, Islamabad, Pakistan; b Farm Technology Group, Wageningen University & Research, Wageningen, the Netherland; c Data Science Institute, National University of Ireland Galway, Ireland

**Keywords:** Hyperspectral imaging, Document image analysis, Document forensics, Writer identification, Ink mismatch detection, Hyperspectral image analysis, Age estimation, Handwritten optical character recognition

## Abstract

This article presents a dataset of hyperspectral images of handwriting samples collected from 54 individuals. The purpose of the presented dataset is to further explore the use of hyperspectral imaging in document image analysis and to benchmark the performance of forensic analysis methods for hyperspectral document images. Each hyperspectral cube in the dataset has a spatial resolution of 512 × 650 pixels and contains 149 spectral channels in the spectral range of 478–901 nm. All the individuals have different personalities and have their writing patterns. The information of age and gender of each individual is collected. Each subject has written twenty-eight sentences using 12 different varieties of pens from different brands in blue color, each approximately 9 words or 33 characters long, all English alphabets in capital and small cases, digits from 0 to 9. The previous methods use synthetic mixed samples created by joining different parts of the images from the UWA WIHSI dataset.Each document consists of real mixed samples written withdifferent pens and by different writers with a variety of mixing ratios of inks and writers for forensic analysis.The standard A4 pages, each weighing 70 gs and manufactured by “AA” company, are used for data collection. The handwritten notes written by each subject with different pens are annotated in rectangular boxes. This dataset can be used for several tasks related to hyperspectral document image analysis and document forensic analysis including, handwritten optical character recognition, ink mismatch detection, writer identification at sentence, word, and character-level, handwriting-based gender classification, handwriting-based age prediction, handwritten word segmentation, and word generation. This dataset was designed and collected by the research team at the Artificial intelligence and Computer Vision Lab (iVision), Institute of Space Technology, Pakistan, and the hyperspectral images were acquired through imaging spectroscopy in the visible wavelength range at Wageningen University & Research, the Netherlands.

## Specifications Table

**Table:** 

Subject	Computer Science; Computer Vision and Pattern Recognition
Specific subject area	Hyperspectral Document Imaging Writer Identification Ink Mismatch Detection Forgery Detection
Type of data	Image Table
How data were acquired	Individuals of the 18–30 age group, who know writing English and have a sense of following complex instructions were given 5 pages data collection form. The completed form is then stored in a safe environment and is sent for hyperspectral scanning. All the collected documents are scanned with Imec SNAPSCAN VNIR hyperspectral camera. Scanning is done with 149 spectral bands of 478.783 nm- 900.972 nm.
Data format	Hyperspectral RAW Images in ENVI Format
Description of data collection	A group of several persons were given instructions and asked for volunteer data collection, 9 of them were selected and instructed accordingly in a single session. All 9 individuals were given 5 pages of the data collection form and are provided with pen # 1. Each participant is followed-up during the data collection process. The data for pen # 1 is collected, when all group members completed the section to be written with pen # 1, the pens were collected back and pen # 2 is provided to each member and vice versa. The mixed combinations for ink mixing are completed in an above-explained manner. For the section to be written by different writers (a mixed combination for writer identification) the documents were shuffled and distributed again. After completion of all the pages, documents were cross-checked and after verification, the document is stored in an envelope holding a tag of “completed documents”.
Data source location	Institution: Institute of Space Technology City: Islamabad Country: Pakistan
Data accessibility	Repository name: Harvard Dataverse Repository Data identification number: https://doi.org/10.7910/DVN/GSYVLD Direct URL to data https://dataverse.harvard.edu/dataset.xhtml?persistentId=doi:10.7910/DVN/GSYVLD

## Value of the Data

• This dataset exploits the usage and possibilities of hyperspectral imaging in document image processing specifically in document forensics.
• This dataset can be beneficial in testing, comparing, and developing different computer vision and image processing classifiers, machine learning, statistical, and deep learning models for document forensics and document image processing.
• This dataset comprises two important niches of digital forensics i.e., Ink and writer. This dataset provides a hyperspectral scan of handwritten text samples that can be utilized for hyperspectral document image processing, optical character recognition, ink mismatch detection, offline writer identification and recognition, age and gender prediction, and word segmentation.
• This dataset consists of 270 hyperspectral images of A4 size stationery paper with a spatial resolution of 512 × 650 and spectral resolution of 478 nm-910 nm resulting in 149 spectral bands.

## 1 Data Description

**Fig. 1:**
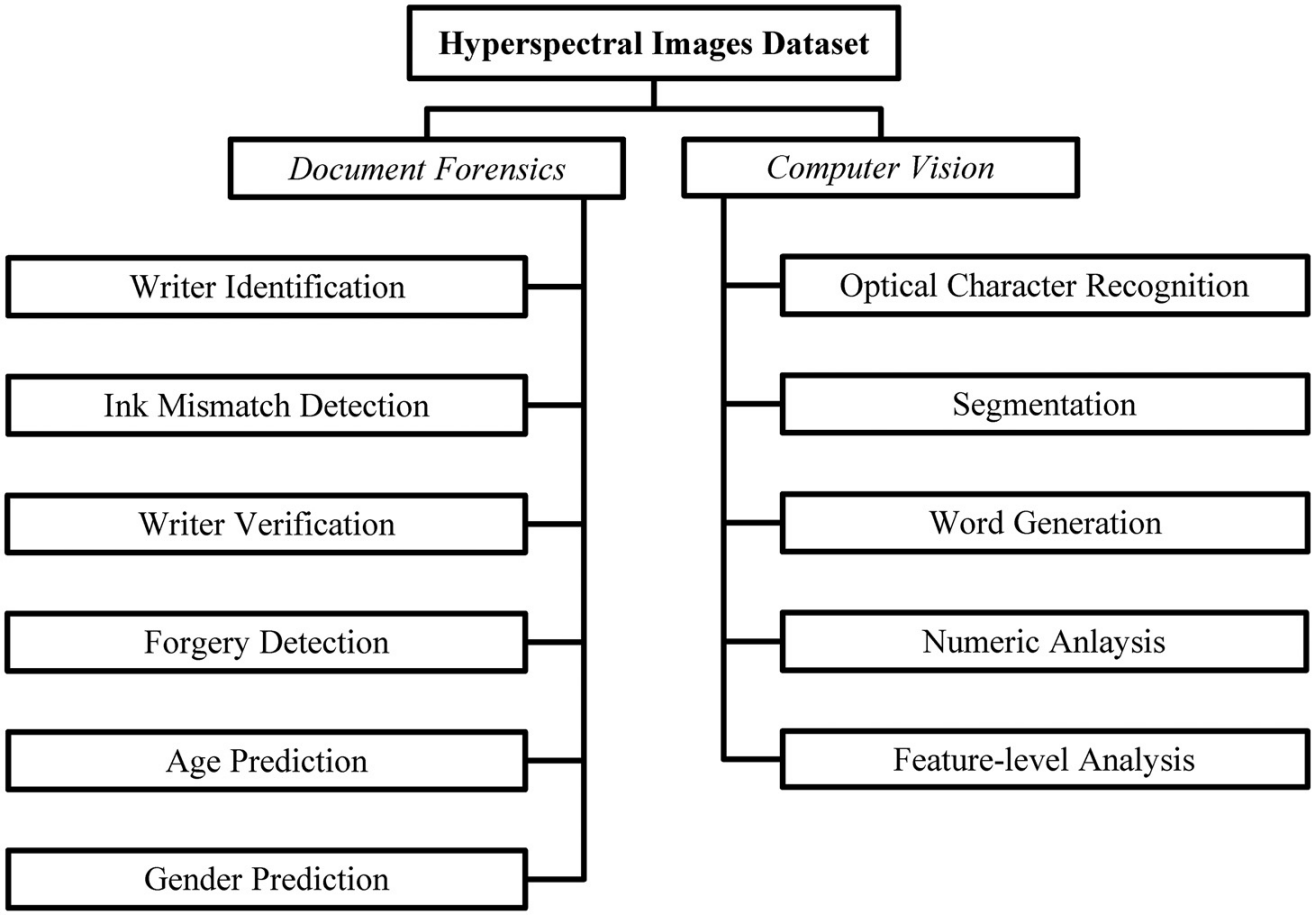
Potential domains of data utilization.

Handwriting and ink are considered to be important features for forgery detection in document forensics [1,2]. Handwriting is considered a potential tool for physiologic modalities of identification, such as DNA and fingerprints [1,3]. Automated forgery detection in document images has quickly evolved due to the digitization of documents [4], common use of hand-held mobile devices, development of advanced sensors and analysis techniques [5]. In recent years, the rich information content of hyperspectral images attracted researchers for its use in ground-based applications [6]. A recent survey article on deep learning-based hyperspectral image analysis shows the potential and wide range of applications of ground-based hyperspectral image analysis in document forgery detection [6]. One of the main problems of using hyperspectral imaging in document forgery detection is the limited number of publicly available datasets [7]. Currently, only one public dataset of hyperspectral images of handwritten samples, named UWA WIHSI is available [8]. The UWA dataset contains the same sentence written by only 7 subjects and it is specifically designed and collected for the ink mismatch detection task. In this dataset, we have collected a large number of hyperspectral images for various document forensic analysis tasks including writer identification and ink mismatch detection. Tables 1 show the brand and manufacturer of pens used for creating this dataset, and the detail of writers is provided in Table 2. In literature [7], [8], [9], [10], [11] mixed combinations were obtained using synthetically mixed samples to validate the efficiency of the proposed technique in more complex scenarios. Keeping the importance of real-time mixed samples, the dataset also comprises real mixed combinations for both writer and ink identification tasks. A different sentence is also included in each document to avoid the biased testing of the techniques and to make it feasible for text-independent handwriting identification methods, as text-independent handwriting techniques show comparatively less accuracy [12]. English alphabets in small and capital cases are written by all individuals. Where each sentence is written by a different pen or writer, alphabets and numbers are separated by a rectangular bounding box. The writer ID, gender, and age are written on the top of every page, while name and signature are collected at the end of page 5. The collected dataset can be used for different document analysis tasks including writer identification, ink mismatch detection, age prediction, gender classification, and handwriting optical character recognition Fig. 1. shows the possible niches where this dataset can be used.

**Table 1:** Manufacturer and brand details of pen/inks used, with pen numbers.

Pen Number	Brand Name	Manufacturer
Pen # 1	Dollar Clipper	Dollar Industries
Pen # 2	Piano Pro	Sayyed Engineers
Pen # 3	Mercury Handy Grip	Mark Industries
Pen # 4	Piano Point	Sayyed Engineers
Pen # 5	Picasso Oria	Shahsons
Pen # 6	Piano Silk	Sayyed Engineers
Pen # 7	Picasso Grip	Shahsons
Pen # 8	Piano Click	Sayyed Engineers
Pen # 9	Piano Click Sky	Sayyed Engineers
Pen # 10	Piano Ball Point Pen	Sayyed Engineers
Pen # 11	Piano Crystal Gel	Sayyed Engineers
Pen # 12	Piano Crystal	Sayyed Engineers

**Table 2:** ID-wise, Age and gender details.

Age	Total	Male ID's	Female ID's	Male	Female
18	5	30,31,42,49	2	4	1
19	16	17,19,20,24,25,27,28,29,32,33,35,36,38,41,46,48		16	0
20	9	18,26,34,37,40,43,44,45,47		9	0
21	6	8,22,50	3,4,6	3	3
22	5	7,13,21,23,39		5	0
24	4	1,15,16	5	3	1
25	2	53	9	1	1
26	3	14,52	10	2	1
27	2	54	11	1	1
30	2	12,51		2	0
	**54**			**46**	**8**

**Table 3:** Quantitative comparison of iVision HHID with the publicly available datasets of hyperspectral handwritten images.

Dataset	UWA WIHSI (Blue)	UWA WIHSI (Blue)	iVison HHID (Proposed)
**Writers**	7	7	**54**
**Inks**	5	5	**12**
**Total Hyperspectral Cubes**	7	7	**270**
**Word Count (per document)**	45	45	**378**
**Character Count(per document)**	165	165	**1465**
**Sentence Count (per writer)**	5	5	**28**
**Numeric Digits**	No	No	**Yes**
**Alphabets**	No	No	**Yes**
**Mixed**	No	No	**Yes**
**Spatial Resolution (Pixels)**	752 × 480	752 × 480	**512** **×** **650**
**Spectral Resolution (nm)**	400–720	400–720	**478–900**
**Bands Count**	33	33	**149**

**Fig. 2:**
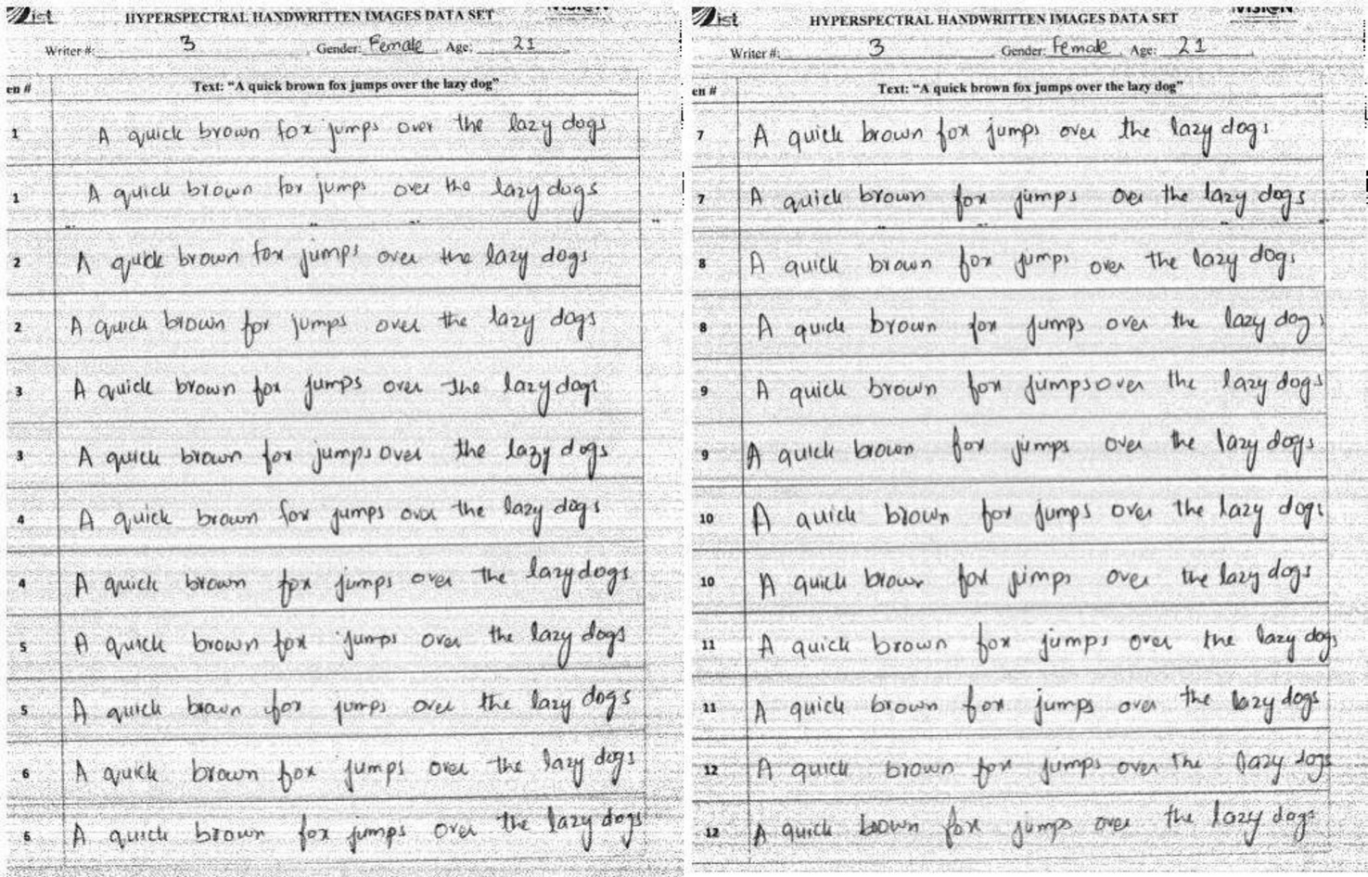
1st two pages of a handwritten document written by Writer#3.

Fig. 2 shows images of the first 3 pages of the document written by Writer #3. These pages are dedicated to the tasks of ink mismatch detection. Each page of the document consists of writer ID, gender, and age on the top. The 1st page comprises the handwritten sample by each writer with six different pens, each pen is used to write two sentences of text “A quick brown fox jumps over the lazy dog”. Similarly, the 2nd page of each document is a collection of the samples with the other six pens. In total, we got 12 sentences on the first page and 24 sentences on both the first and second pages. The identification number referred to as the “pen number” of each ink is written on the left side of each sentence in a separate rectangular bounding box. On the first page, a total of 108 words are collected from each writer, while on both two pages each writer wrote a total of 216 words, 792 characters if spaces are not considered, or 984 characters if space between two words is considered. We collected data from 54 writers, hence we got 108 sentences written with one single pen on page#1 and page#2 of each document.

**Fig. 3:**
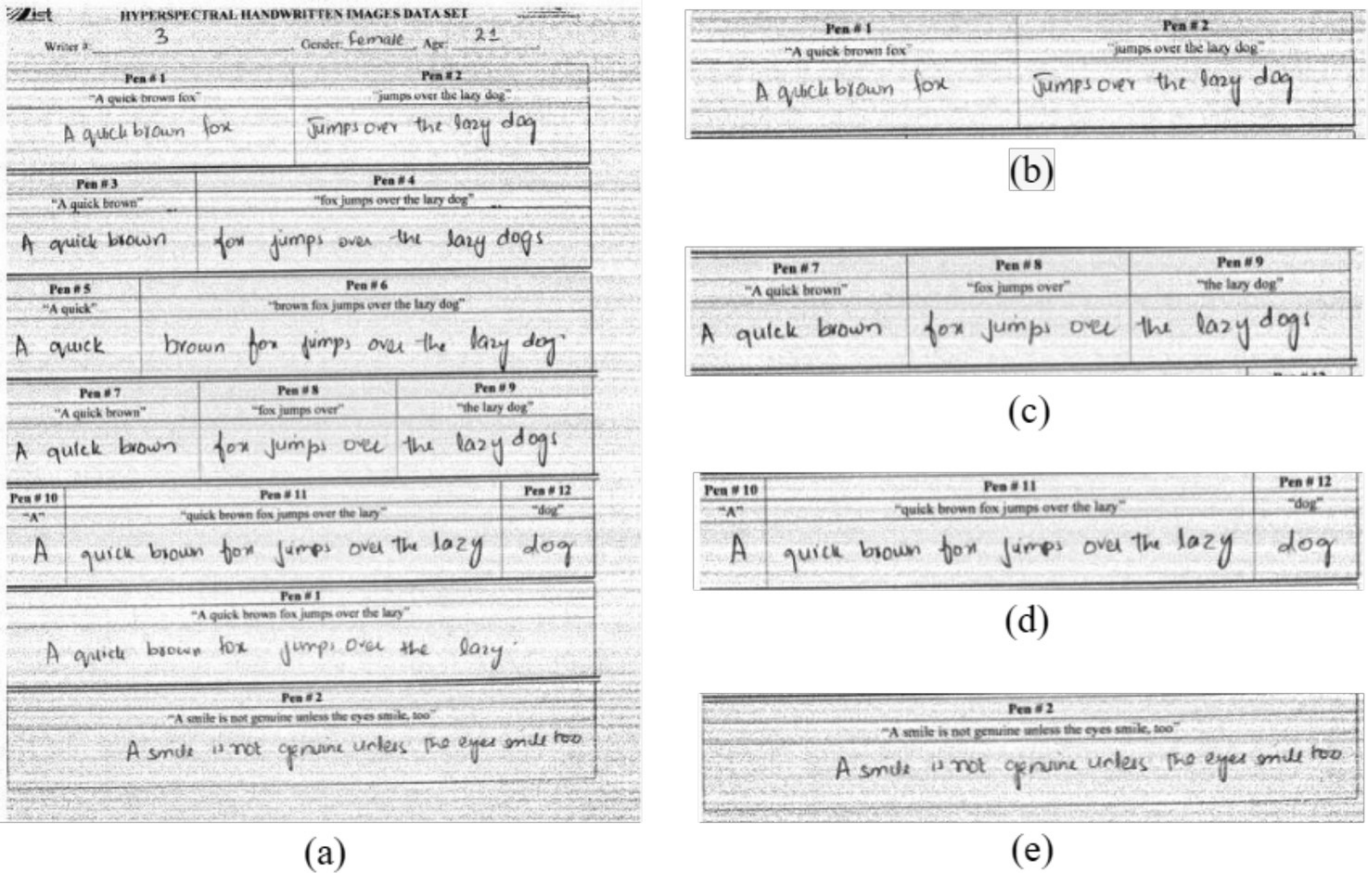
Mixed Combinations of different inks in different ratios(a) 3rd Page written by Writer#3 (b) mixed combination of two inks; Pen#1 and Pen#2 in 1:1 (c) mixed combination of three inks; Pen#7, Pen #8 and Pen #9 in 1:1:1 (d) mixed combination of 3 inks; Pen #10, Pen #11 and Pen #12 in 1:8:1(e) different sentence written by Writer#3 with Pen#2.

Fig. 3(a) shows Page#3 of a document written by writer#3 which consists of the mixed combinations of the text written with different inks in different ratios Fig. 3.(b) is the cropped part of page#3, it shows the mixed combination of two inks in the ratio of 1:1. Similarly, Fig. 3(c) is the mixed combination of three inks in a single sentence written with pen#7, pen#8, and pen#9 in 1:1:1. The identification number of each pen is written on the top of each part of the sentence Fig. 3.(d) is a mixed combination of 3 inks with pen#10, pen#11, and pen#12, the maximum part of the sentence is written with pen#11, resulting in a ratio of 1:8:1. The complete details of the mixed combinations of different inks in the different ratios are given in Table 4. A different sentence to avoid biased testing and to make the dataset feasible for text-independent writer identification and verification techniques is shown in Fig. 3(e).

**Table 4:** Combinations of different writing samples in a single sentence with different ratios.

Number of Writers	Ratio
2	1:1
2	3:7
2	1:4
3	1:1:1
3	1:8:1
4	1:1:1:1
5	1:1:1:1:1
6	1:1:1:1:1:1
9	1:1:1:1:1:1:1

On page#3 total of 63 words are collected from each writer, in which 12 words are written with pen#1, 15 words written with pen#2, 3 words with pen#3, pen#7, pen#8, and pen#9, 6 words with pen#4, 2 words with pen#5, 7 words with pen#6 and pen#11, and one word with each pen#10 and pen#12. Page#3 consists of 235 characters without spaces and 284 characters with spaces written by each writer. These first three pages are designed for ink mismatch detection, where all these collected text samples can be used for writer identification and other relevant tasks shown in Fig. 1.

**Fig. 4:**
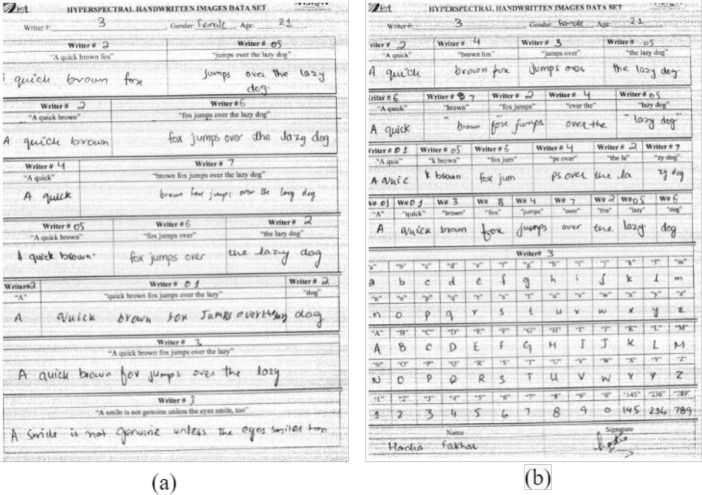
(a)Page#4 of the handwritten document written by Writer#3 (b) Page#5 of the handwritten document written by Writer#3.

**Fig. 5:**
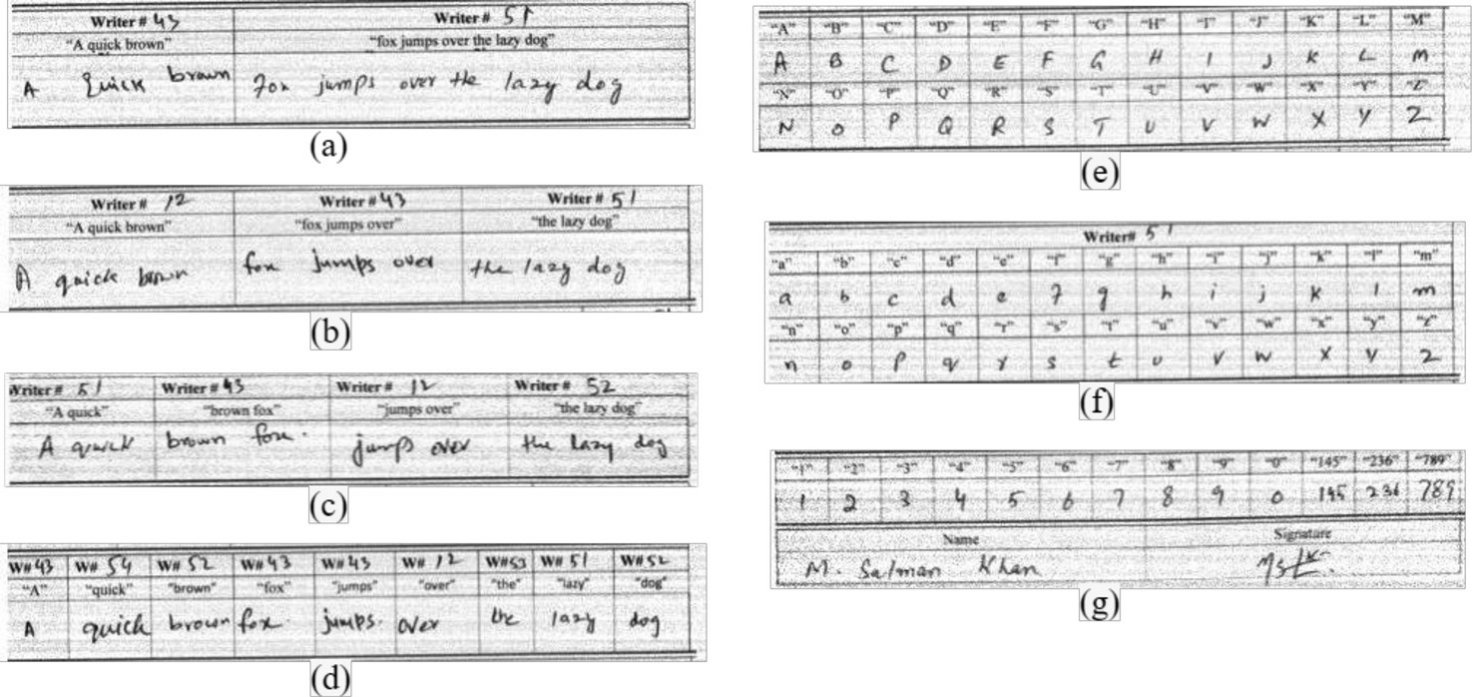
Mixed Combinations of text written by different writers in different Ratios(a) mixed combination of text written by two writers; Writer#43 and Writer#51 in ratio 2:3 (b) mixed combination of text written by three different writers; Writer#12, Writer#43 and Writer#51 in ratio 1:1:1 (c) mixed combination of text written by four different writers; Writer#51, Writer#43, Writer#12 and Writer#52 in ratio 1:1:1:1 (d) Mixed combination of text written by six different writers; Writer#43, Writer#54, Writer#52, Writer#12, Writer#53 and Writer#51 in ratio 1:1:1:1:1:1:1 (e) Capital case English alphabets written by writer#51 (f) Small case English alphabets written by Writer#51 (g) Numeric digits written by Writer#51.

Fig. 4(a) shows page 4 and Fig. 4(b) shows page 5, which is targeted for writer identification. As we got enough samples from each writer by ignoring the ink variable, hence only mixed combinations are collected in this part of the document. Every writer contributed according to the part written on the top of each bounding box. The mixed combinations are collected in different ratios of 2 writers, 3 writers, and up to 9 writers. At the end of each document on page 5, English alphabets in small and capital cases were collected. Numbers from 0 to 9 with three other three-digit numbers were also included. The name and signature of each writer are written at the end of each document Fig. 5. shows the scanned images of Page#4 and page#5. The number of total words collected on page#4 is 63, while the number of characters without spaces are 235 and with spaces 284. On page#5 the total number of words is 36, the number of characters while not counting the space as a character is 203, and the number of characters considering the space is 218. The detailed counting of words, sentences, numerical numbers, and other specifications is shown in Table 1. The details of mixed combinations that are collected from different writers in different ratios are shown in Table 5.

**Table 5:** Combinations of different inks in a single sentence with different ratios.

Number of Inks	Ratio
2	1:1
2	3:7
2	1:4
3	1:1:1
3	1:8:1

**Fig. 6:**
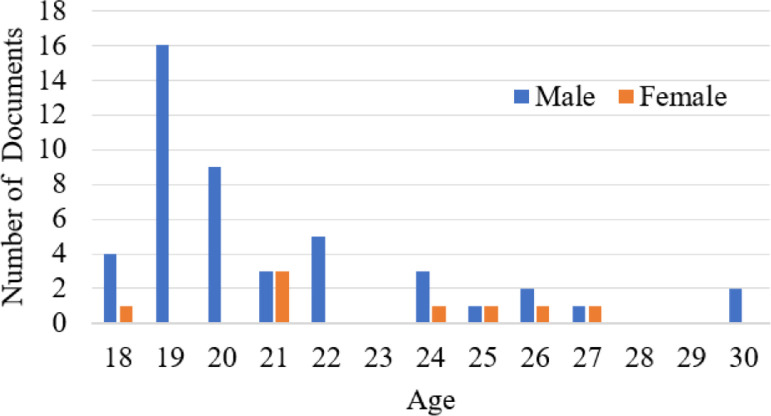
Quantitative distribution, based on age and gender.

The information about age and gender is written on the top of every document. The details of gender with corresponding age group and writer number are given in Table 4. The quantitative distribution of the writers based on age and gender is graphically represented in Fig. 6.

## 2 Experimental Design, Materials and Methods

### 2.1 Data collection

The dataset collection process was carried out at different times in different locations. The writers selected were from educational institutes having a minimum age of 18, all the volunteers were first briefed about the purpose and value of this data and then the volunteers were selected and properly instructed for the filling of the data collection form. A maximum of 9 volunteers was grouped in a single session to ensure the proper guidance throughout the data collection process. All the documents were placed in an envelope tagged “Incomplete Documents”, containing the printed forms with no handwritten text, and the second envelope is tagged as “Completed Documents”. In the first step, all the participants were given the five pages data collection form, then participants were given pen#1. All the pens were properly tagged and only provided to the participants when all of them completed the part to be written with the previous pen. The mixed combination for ink mismatch detection on page#3 of every document was collected in the same manner. For the section to be written by different writers (a mixed combination for writer identification) the documents were shuffled and distributed again. After completion of all the pages, documents were cross-checked and after verification, the document is stored in an envelope holding a tag of “completed documents”.

### 2.2 Data acquisition

The completed documents were further scanned using the hyperspectral camera. The hyperspectral images were acquired using Imec SNAPSCAN VNIR hyperspectral camera. The Spectral scanning of the collected dataset is done with 149 channels of 478.783 nm- 900.972 nm. The total number of documents collected were from 54 writers and each writer wrote 5 Pages. The total number of hyperspectral images scanned is 270.

### 2.3 Data pre-processing

All the scanned images were visualized and checked if any of the important parts of the image were missed during the hyperspectral scanning process. Each image was named properly as w00_p00_corrected.raw. “w00” shows the writer number in place of “00″ and “p00” annotates the page number of each document. To ensure authenticity and quality and the increased size of the data set, data is provided in raw form and no augmentation or processing techniques were applied.

## Ethics Statement

All the handwriting samples were collected from volunteers with their consent, who were priorly informed about the purpose of this data collection.

## CRediT authorship contribution statement

**Ammad Ul Islam:** Conceptualization, Methodology, Formal analysis, Investigation, Data curation, Writing – original draft, Visualization. **Muhammad Jaleed Khan:** Methodology, Investigation, Resources, Writing – review & editing, Project administration. **Muhammad Asad:** Validation, Formal analysis, Data curation. **Haris Ahmad Khan:** Validation, Resources, Writing – review & editing, Funding acquisition. **Khurram Khurshid:** Validation, Resources, Writing – review & editing, Supervision, Funding acquisition.

## Declaration of Competing Interest

The authors declare that they have no known competing financial interests or personal relationships which have or could be perceived to have influenced the work reported in this article.

## Data Availability

iVision HHID (Original data) (Dataverse)
